# Third-Party Access Cybersecurity Threats and Precautions: A Survey of Healthcare Delivery Organizations

**DOI:** 10.1055/a-2713-5725

**Published:** 2025-10-30

**Authors:** George A. Gellert, Daniel Borgasano, Robert Palermo, Gabriel L. Gellert, Sean P. Kelly

**Affiliations:** 1 San Antonio, Texas, United States; 2 Imprivata Inc, Lexington, Massachusetts, United States

**Keywords:** third-party access, cybersecurity, access management, health delivery organizations, information technology, clinical information systems, process improvement

## Abstract

**Objective:**

Gather insights regarding the state of third-party access cybersecurity in healthcare delivery organizations (HDOs).

**Methods:**

An online multinational survey was deployed to eligible respondents to assess HDO third-party access, cybersecurity, and challenges.

**Results:**

Of 209 respondents, only 51.1% reported having a comprehensive inventory of all third parties accessing their network. Sixty percent stated third-party access to sensitive/confidential information was not routinely monitored, despite 19% having more than 40, and 31% having 21 to 40 third parties with network access. Reasons included lack of resources (48%) and centralized control over third-party relationships (36%), complexity (28%), and frequent third-party turnover (22%). Confidence in third-party ability to secure information and their reputations was cited. More than half (56%) reported a breach involving a third party in the last 12 months, and two-thirds anticipate breaches increasing in the next 12 to 24 months. Most agreed breaches are a cybersecurity priority, a resource drain, and their weakest attack surface. Slight majorities indicated high perceived effectiveness in mitigating, detecting, preventing, and controlling third-party access risks and security/privacy regulatory compliance. Regarding existing solutions, roughly half (55%) ranked the effectiveness of vendor privileged access management (VPAM) and privileged access management (PAM; 49%) at ≤ 6 on a 10-point scale, respectively. Barriers to reducing access risks include lack of oversight/governance (53%) and insufficient resources (45%). Of those monitoring third-party access, 53% do so manually. Breach consequences include loss/theft of sensitive information (60%), regulatory fines (49%), severed relationships with third parties (47%), and loss of revenue (42%) and business partners (38%).

**Conclusion:**

HDOs recognize the increasing threat of third-party cyber breaches but are struggling to effectively address them. Lack of budget, expert resources, complexity, and third-party turnover are among the reasons why. Need exists for automated, cost-effective solutions to address the significant risks of third-party access with a consistent strategy that minimizes breach risk by securing remote access to privileged assets, accounts, and data.

## Background and Significance

Healthcare delivery organizations (HDOs) have increased their reliance on external vendors, partners, and contractors, and concomitantly, risks associated with third-party access have evolved into a substantial challenge to cybersecurity. Modern enterprises rely on third parties that include vendors, contractors, suppliers, partners, and supply chain providers for effective operations. However, third parties can be the source of cyber incidents from a successful breach of HDO information systems, or from a third party having excessive access to the HDO network. 1 2 3 4 5 6 7 8 9 10 11 12 13 14 15 16 17 18 19 20

Third parties, such as vendors and contractors, are not HDO employees, but frequently need access, including privileged access, to enterprise devices, systems, applications, and networks. But providing such access creates risks. Third parties are a frequent target of malicious actors because they may have more access than needed operationally, and often have access points into many HDOs (hack one system, breach many). Cyber-attacks commonly involve privileged account compromise, and preventing such malicious breaches through effective privileged access management (PAM) is key to establishing zero trust. 7 8 The global mean cost of a data breach in a survey of 604 organizations was US $4.45 million. 9 It is estimated that 50% of breaches result from stolen or compromised identity credentials, and 61% of organizations reported having experienced a third-party data breach in the prior year. 10 11 Third-party security incidents are more likely to result in regulatory fines than are those caused by internal users). 12

Third parties present unique access management challenges. Not being HDO employees, it is difficult to track their lifecycle and employment/deployment status, and enforce multifactor authentication, or set up their access rights appropriately. Third parties typically do not follow the same governance procedures or identity lifecycle policies, making access difficult to manage and secure. Malicious actors endeavor to leverage third-party access to breach HDO cybersecurity, creating an access management problem for HDOs. 13 14 15 16 17 18 19 20 It is not surprising then that 58% of organizations have no consistently applied access management strategy when it comes to third parties. 12

The attacks of concern are compromises of HDO direct data, system, and network caused by an unauthorized user gaining access to a healthcare customer's network or system via a third-party remote access tool. This could be a virtual private network (VPN) or other method, including remote monitoring and management tools. This is sometimes accomplished via phishing a third-party user and gaining access (usually because multifactor authentication or MFA is not enabled), or by directly exploiting a vulnerability in the core remote access solution to deliver a payload. The type of attack then executed is generally ransomware or data exfiltration. Because third parties that maintain access to healthcare networks are typically of a privileged nature (i.e., an extension of security or information technology [IT] teams), they generally have elevated access privileges to key infrastructure or applications, making such access breaches especially dangerous and attractive to nefarious entities or users.

An illustration of this kind of major data breach occurred in Florida at Broward Health and involved a compromise of the personal and protected health information of more than 1.35 million patients in 2021. 16 A hacker gained access to the Broward Health network through the office of a third-party medical provider who had been granted access to the system's network for providing healthcare services. An investigation found that the attacker had access to parts of the network where employee and patient information were stored, including sensitive identity data, email addresses, telephone numbers, Social Security numbers, financial/bank account information, health insurance information, medical histories, treatment information, medical record numbers, and driver's license numbers. Data was exfiltrated from the Broward Health system. 16 The cyberattack was reported to the U.S. Department of Justice, with subsequent law enforcement investigation. Broward Health improved security in order to prevent similar incidents in the future, including implementing multifactor authentication for all users of its systems and setting minimum security requirements for all devices with access to its network not managed by its IT department. Another major third-party breach occurred in 2023, when Advanced Medical Management, a technical and healthcare management service that offers administrative and technical support to healthcare companies, detected that third-party individuals were on the system network without authorization. It became clear that these were bad actors, hackers intent on stealing valuable personal information. The hackers gained access to the network through a third-party vendor working with advanced medical management. Once inside, they accessed and stole sensitive information, affecting approximately 320,000 individuals, who were consequently at elevated risk of identity theft. 17

The impact of information security breaches on patients is significant. Successful cyberattacks can result in stolen medical records, which include both personal health information (PHI) as well as potentially personally identifiable information (PII). Patient Social Security numbers, insurance and other details can be used for identity theft and other forms of fraud. Medical identity theft involves attackers using stolen health insurance details to obtain healthcare, prescriptions, or medical equipment, potentially leaving victims with inaccurate records and unexpected and unwarranted bills. Cyberattacks can lead to an increase in care complications and patient mortality rates. 21

For HDOs and insurers, reputational harm may result, leading to patient attrition, and reduced revenue and competitiveness. Operational effects on healthcare providers may include care disruptions, when breaches can lead to system downtime or ransomware attacks that lock access to electronic health records, delaying care delivery and risking patient safety. Cyberattacks—and particularly ransomware—can result in critical outages and downtime, which disrupt delivery of critical healthcare services. For example, the ransomware attack in 2024 against Change Healthcare caused widespread outages that resulted in significant delays in care delivery and patients getting needed medications, and delayed health system reimbursement (resulting in some patients having to pay out-of-pocket for care services). 22 The ransomware attack against Ascension in 2024 rendered many patient records inaccessible, causing significant delays in care. 23

Breaches in healthcare are among the most expensive in any industry, generating costs that include the conduct of forensic investigations, breach notification of affected patients, legal fees, regulatory fines, lawsuits, and remediation efforts. The average cost of a data breach in healthcare is US $7.42 million, the highest of any industry for the 14th consecutive year. 24 Healthcare breaches require the longest amount of time to identify and contain (a mean of 279 days), which is 5 weeks longer than the global average. 25 In addition, losses in productivity occur as a result of information security breaches, as hospital or health system IT and compliance teams must divert resources to manage the incident response, and clinical staff may need to revert to manual processes during the downtime period.

The three pillars of establishing third party security are (1) PAM to secure the credential/account; (2) access governance to ensure that third parties have the right minimal level of access rights for their role; and (3) secure network access that maintains third parties abstracted from the physical network and isolated to prevent lateral movement. 3 PAM and vendor PAM (VPAM) systems typically have multi-layered security measures. Common features often include zero trust network access, which limits access to specific protocols/ports and hosts to prevent lateral movement within an organization's information system. Data is fully encrypted in transit and at rest. Table 1 identifies key features of VPAM and PAM. Strong authentication and access approvals are typically required, such as MFA and additional steps before access is granted, including access schedules enabling access during specified days and times. Access notifications are sent to security or IT to notify them of access, and/or access approvals by a member of IT or security are required before granting access. Employment verification validates that users are current employees of the vendor/third party. Session monitoring occurs via keystroke and video audit of sessions, and detailed metadata about each connection is collected. Credential vaulting is used to ensure that privileged credentials are encrypted and stored in a software vault, and users must request to check out credentials. Credentials can also be set to be randomized/rotated at a regular interval to reduce the likelihood of a compromised credential being useful at a later time. Tables 2 and 3 summarize how VPAM tools support Office of Civil Rights (OCR) audit readiness and General Data Protection Regulation (GDPR) compliance, respectively.

**Table 1:** Key features of VPAM and PAM

Zero trust enforcement • Enforces least privilege, giving vendors access only to the specific systems/services they need • Outbound-only, brokered connections—no VPN tunnels or direct inbound traffic • Continuous verification via MFA, SSO, identity federation (SAML/LDAP/AD) • Can further provide granular access controls like time limits, access schedules, and access approvals • Enforces just-in-time privilege elevation (no standing admin rights) • Requires MFA and granular authorization for every privileged session
Credential vaulting • Vendors or internal users need never be exposed to system passwords • Credentials are stored encrypted, rotated, and hidden • Eliminates shared/generic administrative accounts
Employment verification • A roster of reps is kept associated with a vendor entity at all times • Reps associated with a “Nexus Vendor” (i.e., a vendor using Imprivata themselves to provide remote support), the rep is locally authenticated with their company's IdP • All other reps/users are verified to have access to a valid corporate email address at the vendor they are associated with prior to each connection
VPAM/PAM supports NIST SP 800-207 tenants of zero trust by making every access by a vendor explicitly authenticated, authorized, encrypted, and monitored • VPAM treats every system as a controlled resource, tightly scoped by policy • Connections are limited to a host and service needed—no broad-based VPNs • Connections are brokered outbound only and encrypted • No implicit trust for “internal” vs. “external” access—all connections are limited, controlled, and audited in the same manner • Vendors cannot be persistently connected, but will time out, and the system can enforce access schedules or time limits • MFA and employment verification can be required before each connection • Session recording and audit logs support continuous monitoring and logging
VPAM/PAM includes additional features to support ISO 27001 • Can enforce governance policies in line with organizational security policies • Uses employment verification steps to ensure only active, verified vendor reps can connect • Vaults and manages credentials to help manage secret authentication information • Supports supplier oversight by enforcing access approvals

Abbreviations: ISO, International Organization for Standardization; PAM, privileged access management; VPAM, vendor privileged access management.

**Table 2:** How VPAM supports OCR audit readiness

Least-privilege, time-bound vendor access (access controls) • VPAM scopes access down to specific hosts/ports (“Applications”), with role/date/time rules—avoiding broad VPNs. This aligns to 164.312(a)(1) and least-privilege expectations
Strong authentication and identity (person/entity authentication) • Integrates with SAML/AD/LDAP for unique identities and MFA; avoids shared accounts
Comprehensive audit trails and session evidence (audit controls) • VPAM records detailed session history, per-service logs (SSH/Telnet/FTP/Desktop Sharing), and can capture full-HD session recordings/command logs—showing who connected, when, to what, and what they did. Logs can be exported or sent to your SIEM via syslog
Centralized vendor inventory and approvals (administrative safeguard support) • Maintains a current roster of vendors and individual reps, with approvals and connection forms—useful when OCR asks how you govern business associates and their workforce
Encrypted connectivity and segmented design (transmission security) • Gatekeeper architecture uses outbound-only connections and encrypted tunnels, limiting network exposure while protecting sessions in transit (164.312(e))
Prebuilt reports for audits • Built-in reports let you quickly generate user activity and service-usage reports for defined periods (e.g., “last 90 days”)

Abbreviations: OCR, Office of Civil Rights; VPAM, vendor privileged access management.

**Table 3:** How VPAM supports GDPR compliance

Access control and data minimization (Art. 5, 25, 32) • VPAM enforces least privilege by scoping access down to only the specific systems, services, and timeframes vendors require • No open VPNs or shared credentials—every action is purpose-limited and attributable to an individual
Strong authentication and accountability (Art. 5(2), 28, 32) • Supports unique identities via SSO, SAML, AD/LDAP, and MFA • Eliminates generic/shared vendor logins, strengthening accountability • Helps satisfy controller–processor contract obligations (Art. 28) by proving you enforce technical safeguards over processor access
Audit trails and monitoring (Art. 30, 33) • Detailed session logging (timestamps, commands, keystrokes, video recordings) • Evidence supports records of processing activities (Art. 30) and incident investigations (Art. 33/34) • Logs can be exported to SIEM for monitoring, anomaly detection, and incident response documentation
Data security and confidentiality (Art. 32) • All connections use encrypted tunnels (TLS) • Gatekeeper architecture ensures outbound-only vendor connectivity, reducing exposure • Supports network segmentation, helping limit lateral movement in case of compromise
Accountability and demonstrability (Art. 5(2), 24) • Provides audit-ready reports that demonstrate compliance with GDPR principles • Enables policy enforcement documentation (e.g., vendor onboarding, approval workflow) • Simplifies proving to regulators or auditors that processor/vendor access is appropriately governed

Abbreviation: VPAM, vendor privileged access management.

Assigning the correct level of privileged access is critical to prevent security incidents and to ensure third parties and employees have sufficient access in order to be productive. A comprehensive, consistent access strategy can minimize cybersecurity risk by securing remote access to privileged assets and accounts, improving security without sacrificing productivity, and saving time. 10 11 12 13 14 15 16 17 18 19 20 This is especially critical for third-party access, which is both heavily targeted by bad actors and less scrutinized than internal users.

Recent issuance of new mandates includes the U.S. Federal Trade Commission (FTC) Health Breach Rule, which is designed to extend beyond HIPAA-covered entities. VPAM is not limited in its scope, can be (and is) deployed by organizations to manage vendor access and activity, including those that may not fall under HIPAA but would be subject to the U.S. FTC Health Breach Rule. Like its support for an OCR audit or breach for a HIPAA-covered entity, VPAM can limit data exposure, help identify the scope and root cause of a breach, and help support the FTC breach notification rule by providing rapid, detailed context. VPAM can also help address OCR AI guidance by granting granular time-bound access to only the systems and services where AI vendors need help to support data minimization and purpose limitation. VPAM provides a comprehensive history and audit trail of all activity, and in the event an organization would need to explain how AI was used and by whom, VPAM could support this transparency.

In sum, the modern HDO enterprise relies on third parties—including vendors, contractors, suppliers, partners, and broader supply chains—to operate effectively, yet those same third parties are frequently the source of cyber incidents, whether it be a successful system breach or a third party having too much access to an HDO network. Given the critical importance of third parties to HDO operations, and little data reported on health system preparedness and effectiveness in managing third-party access, this survey sought to understand current HDO third-party cybersecurity precautions, practices, capabilities and perceived challenges in effectively managing third-party access to their network, information systems, and sensitive data.

## Objectives

To gather information in four nations about the practices and perceptions of HDOs regarding precautions to ensure the cybersecurity of third-party access to information systems and sensitive/confidential data and to assess the pervasiveness of third-party access risk, the effects of third-party related breaches, and the challenges faced in managing the breach threat from third-party access.

## Methods

### Study Design and Setting

An online fully de-identified and anonymous survey was deployed to IT and IT cybersecurity personnel in HDOs, including health systems and hospitals. The survey was created and pretested by Ponemon Institute and Imprivata. Data was extracted from a larger 2024 multisector survey of 1,942 respondents on third-party cybersecurity in the healthcare, government, financial services, and industrial/manufacturing sectors. 12 A delimited analysis of data from 209 (10.8%) respondents who reported having an IT cybersecurity management and/or leadership role was completed. The analyses sought to understand organizations' approaches to managing cybersecurity, with a focus on managing third-party remote access risks.

### Data Captured and Analyses Completed

Survey questions gathered information about respondents' organizational role(s) and evaluated the current status of and gaps, concerns, needs and challenges in third-party access cybersecurity management. Respondents were sought from four nations: Australia, Germany, the United Kingdom, and the United States. Survey responses were quantified and tabulated. Survey data was analyzed using stratified contingency tables, and bar chart histograms were generated to share the response results differentiated by nation. A listing of the questions used in the survey is available ( Supplementary File , available in the online version only)

### Institutional Review Board Approval

This study did not involve patients or patient data, and the survey of healthcare IT personnel was completed on an opt-in basis where respondents explicitly agreed to have their data analyzed and published in aggregate, de-identified form. As such, IRB approval and formal ethical review were not needed or pursued.

## Results

### Care Setting and Respondent Characteristics

Respondent organizations included hospitals, outpatient care settings, home health, behavioral health, and skilled nursing facilities. Fifty percent worked in facilities with a headcount of less than 5,000 staff, 23% had 5,001 to 25,000 staff, and 27% had greater than 25,000 staff. Eligibility criteria for survey participation validated that respondents were leaders in managing enterprise IT cybersecurity and were familiar with their organizations' approach to managing privileged access, including processes and technologies used to secure privileged access for third parties at their respective organizations. With respect to organizational position, 30% of respondents were either vice presidents, senior executives or directors, and 31% were supervisors, managers, or technicians. Twenty-two percent characterized themselves as staff. Twelve percent of respondents were in Australia, 30% were in Germany, 20% in the United Kingdom, and 38% in the United States. Germany and the United States were overrepresented due to a potential sampling bias in national response rates. This distribution is not proportional to population size and may possibly skew regional insights. However, our purpose in describing the international distribution of survey responses is less to draw insights about the particularities of third-party access risk between nations than it is to illustrate that cybersecurity concerns and issues transcend borders and are common in HDOs attaining a certain level of health informatics development, wherever located.

The total sample size of completed HDO surveys was 209. Meaningful differences in specific question responses stratified by nation were not apparent. Respondents reported annual IT cybersecurity budgets as follows: 26%—US $25 million per year or less; 26%—between US $26 and US $100 million per year; 32%—US $101 to US $500 million per year; and 16% in excess of US $500 million annually.

### Organizational Strategy and Current Practices for Managing Third-Party Access Risks

Respondents were asked about their organizational approach to managing privileged access abuse, including processes and technologies used to secure third-party access, and whether the HDO employed a solution for VPAM or PAM. Thirty-four percent indicated their HDO employed both VPAM and PAM, 35% employed only VPAM, and 31% only PAM. Nineteen percent of HDOs reported greater than 40 third parties and vendors with access to the organization's network; 31% reported 21 to 40 third parties/vendors with access; 41% reported 1 to 20; and 9% reported none. On average, 22 third parties and vendors had HDO network access. Only 51.1% of HDOs reported having a comprehensive inventory of all third parties with network access. HDOs not having a third-party inventory stated it was due to a lack of resources to track third-party access (48%); the HDO had no centralized control over third-party relationships (36%); 28% cited complexity and 22% had a high frequency of turnover of third parties.

Fifty-seven percent of HDOs did not evaluate the security and privacy practices of all third parties before providing access to sensitive or confidential information. Sixty percent reported that third parties with access to confidential information were not routinely monitored. Of those who do monitor third-party access, 53% do so manually.

### Third-Party and Vendor Data Breach and Cyberattack Experience

More than half (56.4%) of HDOs reported a breach involving a third party accessing their network in the last 12 months. Misuse of HDO sensitive or confidential information resulted in 52.7% of known instances, and in 54.3% the data breaches or cyberattacks were thought to be the result of a third party having too much privileged access. Further, 48% of organizations believe that third-party remote access is becoming their weakest attack surface, and it comes with consequences, including loss or theft of sensitive and confidential information, regulatory fines, and other operational disruptions. Two-thirds of HDOs anticipate the number of data breaches caused by third parties will increase in the next 12 to 24 months, and 24% expect the level to remain the same. The mean cost of restoring access to third-party and privileged access users over the prior 12 months was US $86,550.

### Perceived Effectiveness and Maturity of VPAM Solution

Perceptions varied highly regarding how effective the organization's VPAM solution is in reducing privileged access abuse: 55% ranked effectiveness at six or below on a 10-point scale, where 10 is maximal effectiveness; 31% ranked at 7 to 8, and only 14% at 9 to 10. For PAM, 49% ranked their solution effectiveness at six or below, with 33% at 7 to 8, and 18% at 9 to 10. Only 36% indicated that their strategy to address privileged access risks is applied consistently, and the balance stated their application was inconsistent, ad hoc, informal, or nonexistent. HDOs ranked their perception of third-party effectiveness in compliance with security and privacy regulations on a 10-point scale: 47% ranked at six or below, and only a fifth (22%) ranked at 9 to 10.

### High-Level Third-Party Cybersecurity Challenges and Barriers Facing HDOs

HDOs reported the challenges facing their organization in managing the cybersecurity of third-party privileged access, shown in Table 4 . Near majorities strongly agreed or agreed that third-party data breaches are increasing, are a cybersecurity priority, and a resource drain.

**Table 4:** Increasing challenges of managing the cybersecurity of third-party privileged access

	Third-party and vendor data breaches are increasing	Third-party remote access is a cybersecurity priority	Managing third-party permissions and access is a resource drain	Third-party remote access is the weakest attack surface	Have visibility into users' level of access
Strongly agree	28%	21%	25%	19%	28%
Agree	19%	25%	20%	25%	23%
Unsure	15%	26%	20%	21%	21%
Disagree	17%	8%	19%	15%	14%
Strongly disagree	15%	20%	16%	20%	14%

HDOs reported barriers they face in reducing third-party and privileged access risks, and most frequently cited a lack of oversight or governance (53%) and insufficient resources (45%; Table 5 ).

**Table 5:** Barriers to reducing third-party and privileged access risks

Barrier to reducing third-party access risk	Percent responding
Insufficient resources/budget	45%
Insufficient visibility of people and business processes	43%
Insufficient assessment of risks	40%
Difficulty in hiring and training; lack of skilled personnel	37%
Lack of leadership	42%
Lack of oversight or governance	53%
Complexity of compliance and regulatory requirements	35%

### HDO Actions and Organizational Processes to Ensure Appropriate Third-Party Access

Table 6 identifies the efforts HDOs have implemented to ensure appropriate third-party access to high-sensitivity data assets. Majorities cited utilizing network segmentation/isolation, removal of access credentials, and verification of third-party need to access.

**Table 6:** Efforts to ensure appropriate third-party access to high-value data assets

Actions to ensure appropriate access	Percent responding
Enhanced physical controls (i.e., restricted control areas)	36%
Restriction of network access	46%
Enhanced identity and access management techniques	29%
Ensure access entitlement is appropriate to the job function	45%
Network segmentation/isolation	69%
Remove access credentials when appropriate	66%
Verification of a third party needs to have network access	56%
Education of privileged users	44%
Processes to prevent abuse of privileged access	
Perform background checks before issuance of privileged credentials	57%
Conduct manual oversight	56%
Monitor and review provisioning systems	51%
Review and act upon threat intelligence	46%
Deploy identity access and management policy monitoring tools	47%
Conduct regular privileged user training	41%

Many HDOs had not engaged in basic precautions and processes to prevent abuse of privileged access ( Table 7 ).

**Table 7:** HDO perceived effectiveness in managing third-party access risks

	Mitigating third-party remote access risks	Detecting third-party remote access risks	Preventing third-party sharing of access credentials	Controlling third-party access to the organization's network	Third-party compliance with security/privacy regulations
Effectiveness 10-point scale					
1 or 2 (low)	12%	12%	12%	11%	11%
3 or 4	14%	13%	15%	13%	14%
5 or 6	22%	25%	20%	20%	22%
7 or 8	32%	30%	32%	35%	31%
9 or 10 (high)	20%	20%	21%	21%	22%

Abbreviation: HDO, healthcare delivery organization.

### HDO Perceived Effectiveness in Managing Third-Party Access Risks

Only slight majorities of HDOs were generally confident about their perceived effectiveness in managing third-party access risks—in mitigating, detecting, preventing, and controlling critical third-party access breaches and achieving compliance with security and privacy regulations ( Table 8 ).

**Table 8:** Challenges in granting and enforcing privileged user access rights

Challenge in granting and enforcing privileged access	Percent responding
Takes too long to grant access to privileged users	61%
Too expensive to monitor and control privileged user access	60%
Too many staff are required to monitor and control privileged users	53%
Cannot apply access policy controls at the point of a change request	70%
Timing of granting access to privileged users is staggered	62%
Cannot keep pace with the number of access change requests	56%
Lack of a consistent approval process for access	38%
Difficult to audit and validate privileged user access changes	28%
Burdensome process for business users requesting access	24%
No common language exists for access requests to IT and business units	40%

### HDO Challenges in Granting and Enforcing Privileged User Access Rights

HDOs reported that the time and costs required to grant, monitor, and enforce privileged user access rights, and the inability to apply access policy controls at the point of a change request, were major challenges ( Table 9 ).

**Table 9:** Required security and privacy practices before conveying third-party access

Required security and privacy practice	Percent responding
Third-party reviews HDO's written policies and procedures	56%
Contractually obligates the third party to adhere to security and privacy practices	48%
Obtain indemnification from the third party in the event of a data breach	39%
Assess third parties' security and privacy practices	51%
Obtain a self-assessment completed by the third party	41%
Obtain references from other organizations that engaged the third party	32%
Obtain evidence of security certification, such as NIST ISO 2700/27002 or SOC	18%

Abbreviations: HDO, healthcare delivery organization; ISO, International Organization for Standardization; NIST, National Institute of Standards and Technology; SOC, security operations center.

### Required HDO Security and Privacy Practices for Third-Party Access

Table 10 shows HDO security and privacy practices required when granting third-party access, and demonstrates how infrequently many of these are applied.

**Table 10:** Reasons security and privacy practices are not engaged before and after conveying third-party access to sensitive and confidential information

Reasons security and privacy practices not engaged	Prior to conveying access (percent responding)	After conveying access (percent responding)
High confidence in the ability of a third party to secure information	63%	61%
Reliance on the business reputation of a third party	56%	56%
Inadequate internal resources to check or verify	58%	48%
Have insurance limiting liability in the event of a data breach	45%	44%
Third party is subject to data protection regulations that protect information	41%	29%
The third party is subject to contractual terms	32%	23%
The data shared with the third party is not sensitive or confidential	34%	26%

### Reasons Security and Privacy Practices Are Not Engaged with Third Parties

Table 11 illustrates reasons why HDOs do not engage in security and privacy practices prior to and following granting access to a third party.

**Table 11:** Actions taken to ensure third-party compliance with privacy and security regulations

Identify and categorize third-party vendor and partner access needs	58%
Perform access assessments for each vendor and partner	57%
Encrypt transmissions for all open or public networks	51%
Implement least privileged access	51%
Insist on unique user access credentials	50%
No vendor-supplied security parameters or default passwords	49%
Develop secure application and system implementation	44%
Capture detailed audit logs of each support session	36%
Install and maintain a firewall configuration to protect data	35%
Track and monitor all access to network resources and critical	35%
Restrict physical access	35%
Protect all systems against malware and regularly monitor anti-virus protection	33%

### Actions Taken to Ensure Third-Party Compliance

HDOs endeavor to ensure third-party compliance with privacy and security regulations, and are identifying and categorizing third-party vendor and partner needs, completing access assessments for each of the latter, encrypting transmissions for open and public networks, and implementing least privileged access. However, only slight majorities of HDOs engaged these measures ( Table 11 ).

### Consequences of Data Breaches and Cyberattacks

**Fig. 1:**
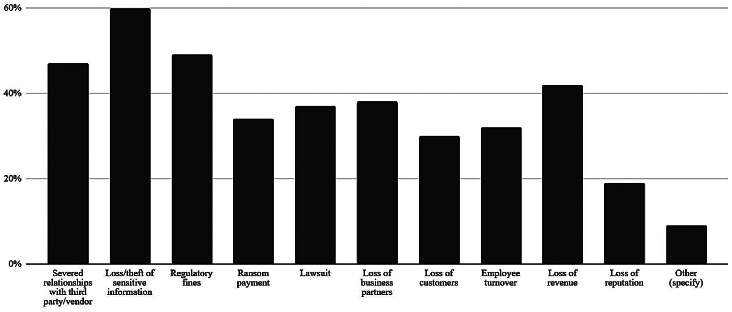
Consequences of data breaches and cyberattacks.

HDOs reported substantial consequences of data breaches and cyberattacks, as shown in Fig. 1 , including loss or theft of sensitive information (60%), regulatory fines (49%), severed relationships with third parties (47%), and loss of revenue (42%) and business partners (38%).

### Time Expended Investigating Third-Party and Privileged Access Security

**Fig. 2:**
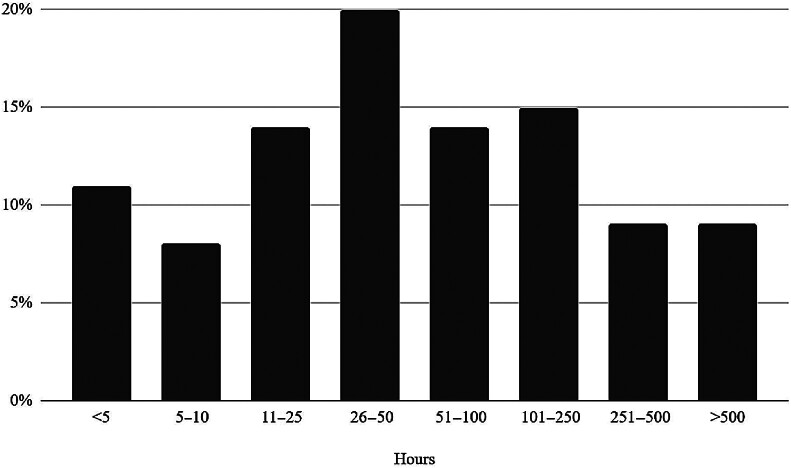
Hours per week expended investigating third-party and privileged access security.

Twenty percent of HDOs expend 26 to 50 hours per week investigating third-party privileged access security concerns, and 15% spend 101 to 250 hours; this is followed by 11 to 25 hours and 51 to 100 hours spent per week. Only 19% of HDOs reported spending 10 or fewer hours per week on these issues. Eighteen percent spend 251+ hours per week, with half of those spending more than 500 hours per week. On average, HDOs expend 129 hours per week analyzing and investigating third-party privileged access issues and concerns. ( Fig. 2 )

## Discussion

The survey data identifies a troubling pattern of HDO management of third-party access and cybersecurity precautions, with just half of respondents having reported the completion of a comprehensive inventory of third parties with network access. This was largely due to a lack of tracking resources, not having centralized control over third-party relationships, task complexity, and high frequency of third-party turnover. More than half of HDOs experienced an access breach involving a vendor or other third party in the last year, and believe that third-party access is becoming their weakest attack surface, causing loss or theft of sensitive information, regulatory fines, operational disruptions, severed relationships with third parties, and loss of revenue and business partners. Two-thirds anticipate third-party breaches increasing over the next 2 years.

Many HDOs had not engaged basic precautions and processes to prevent abuse of privileged third-party access, and are not confident about how effectively they are managing their organizational risks, with only a slight majority indicating perceived high effectiveness of current practices. A majority do not evaluate the security and privacy practices of third parties before enabling access to sensitive or confidential information, and do not routinely monitor these practices. Among those who do monitor third-party access, most do so manually. Among the minority of HDOs that have deployed VPAM or PAM, almost half had modest or low confidence in their deployment of these solutions and reported mediocre achievement of compliance with security and privacy regulations. Two-thirds indicated that their strategy to address privileged access risks is applied inconsistently. Yet, third-party related cybersecurity breaches can directly impact not only patient care, but also the supply chain that is essential to all care delivery operations. 19

The primary reasons why PAM and VPAM may underperform include, first, that these solutions are deployed minimally or only partially by HDOs. Organizations may, due to budget or resource constraints, deploy a PAM solution to a minimal versus an optimal extent. Often this includes only basic credential vaulting capabilities, which are usually completed to comply with an imposed mandate (e.g., internal security-driven, cyber insurance, etc.). Also, internal expertise or resource availability may be limited within those organizations that do deploy enterprise-grade PAM capabilities. Frequently, organizations suffer from unrealized value due to long, complex implementations, and/or ongoing customization requirements or generally cumbersome ongoing administration to support the solution. Lack of third-party specific tools is another challenge, in that often standard PAM tools are not third-party specific, and do not include the needed extensions to handle third-party users, including secure remote access, the ability to enforce MFA on third-party users, and employment verification.

Reasons for lax third-party cybersecurity precautions and practices included HDOs having high confidence in the ability of third parties to secure information and a reliance on the business reputation of the third party. Common barriers to reducing third-party and privileged access risks included a lack of oversight or governance, insufficient resources/cost, the amount of time required to grant and monitor access of privileged users, and the inability to apply access policy controls at the point of a change request. Methods that HDOs can deploy to improve third-party access security include implementing the principle of least privilege, which grants users the minimum access required to perform their duties, regularly auditing access, and conducting periodic reviews to identify and revoke unnecessary permissions. 13 14 15 In addition, educating users on security best practices and about the importance of data protection and responsible access management remains central. 13 14 15 16 17 18 19 20 26 27 Blanchard et al reported on the successful implementation at scale of a tabletop cybersecurity simulation that enabled health informatics trainees opportunities to apply knowledge related to cybersecurity breaches to real-world examples. 26 In terms of how best to target such training, research reported by Alhuwail et al suggests that nurses demonstrate higher cybersecurity aptitude relative to physicians, and physicians in particular need to have protected time set aside to receive adequate cybersecurity training, despite the opportunity costs and other issues competing for their attention. Further, professionals who were less inclined to use the internet for personal use during their work time demonstrated a higher cybersecurity aptitude. 27 Technology enhancements would include tools that provide more deterministic workflows and capabilities. Many larger vendors offer a great deal of customization, when in many cases, a best practice/standard deployment would be more effective and cost-effective. Additionally, the industry is moving toward more integrated platforms of access management solutions (of which privileged access is one component). This results in less overhead and conveys the benefits of a single system to manage all user classes, whether privileged, third party, employee, or patients.

HIPAA requires healthcare organizations to maintain information security and confidentiality compliance by demonstrating a high level of visibility and control around business associates' access to critical systems and patient data. Traditional remote access tools, such as VPNs or desktop sharing, do not meet the safeguard standards required to pass an internal or U.S. OCR audit. These tools are not designed to restrict access only to authorized users, nor do they provide visibility through recording all access activity for review and examination. A VPAM solution can help ensure compliance with HIPAA security rules by maintaining policies and procedures to comply with—and document compliance with—requirements. A single system can ensure adherence and provide transparent documentation that all access to PHI complies with regulatory requirements. HDOs across nations need to implement procedures to regularly review records of information system access and activity, with a single bespoke system where all activity is logged and can be reported on, thus providing one source of truth for all activity reports and audit logs. Further, regulatory imperatives across nations require that HDOs ensure that electronic PHI (ePHI) is not altered or destroyed, which can be ensured via recording of all information system access and activity by third parties. The global standard emerging is that HDOs should be able to verify that a person or entity seeking ePHI is the one claimed, which can be achieved via MFA and employment verification.

It is imperative to ensure sufficient resources, in-house expertise and technologies exist to improve the efficiency and security of access governance processes, and to keep pace with the number of access change requests while reducing burdensome processes for third parties and business users requesting access. 13 14 15 16 17 18 19 20 Automating processes involved in granting privileged user access, and reviewing and certifying privileged user access to meet growing requests for access changes, are essential best practices, as is maintaining an inventory of third parties and internal users with privileged access. Without such inventories, HDOs cannot gain a unified view of privileged user access across the enterprise. 13 14 15 Regarding the paucity of resources available for improving third-party access security, quantitative detailing for HDO non-IT leaders about the potential financial downside caused by a potential breach as a result of third-party access, with a cost-benefit analysis, may help in obtaining the resources necessary to invest in improving precautions.

In order to manage third-party cybersecurity challenges effectively, the technology solution deployed needs to achieve a net reduction of overhead costs for HDOs. Purpose built solutions for managing third party access have emerged which automate critical manual tasks that are not easily accomplished using standard PAM or identity governance administration and access management tools. Needed solution capabilities include managing third-party roles and automatically assigning access rights, automatically onboarding and deprovisioning third party users, providing safe and secure remote access and a single source for all third-party activity to accelerate the process of responding to audits or investigating an incident. Automated, cost-effective solutions should come with out-of-the-box (OOTB) vendor access capabilities that include remote access, employment verification, native MFA options, and the ability to create and define “vendor” entities and roles to avoid more integration work and frequent manual steps. Solutions should come OOTB with HIPAA compliance mapping and other compliance frameworks. Best-of-breed solutions should automatically notify IT administration if any system changes implemented cause the configuration to fall out of selected compliance frameworks. They should offer cloud SaaS and should optimally provide relatively deterministic workflows for HDOs that can be configured via the user interface without the need for intensive services to implement or make changes to the system. Ideal solutions should tackle all the key problems identified in the survey without requiring custom integrations, configuration, heavy training, etc., so that HDOs secure third-party access while reducing IT administrative burden/lift and net overhead expenditure. Technologies exist that engage a zero trust remote access method to prevent potential lateral movement and limit blast radius if there were an access breach by a nefarious user.

Study limitations include the fact that when surveys are sent to a representative sample of individuals, and despite nonresponse tests, individuals who did not participate may be substantially different in terms of potential responses from those who completed the survey, causing potential nonresponse bias. Sampling-frame bias is another potential limitation, in that the respondents selected for participation may not be representative the broader group of individuals who are IT and IT cybersecurity practitioners. Further, because a web-based data collection method was used, it is possible that non-web responses by mailed or telephone survey might have yielded different findings. Self-reported results are also based on the integrity of confidential responses received from subjects, and while checks were incorporated into the survey process, it is possible that not all subjects provided accurate and truthful responses.

## Conclusion

HDOs have increased their reliance on external third-party vendors, partners and contractors, and concomitantly, risks associated with third-party access have evolved into a substantial cybersecurity threat. Challenges HDOs face in effectively addressing the threat include a lack of budget and expert resources, complexity, and third-party turnover. HDOs are lacking and need a comprehensive, consistent access strategy to minimize third-party cybersecurity risk by securing remote access to privileged assets so as to improve security without sacrificing productivity. This is especially critical for third-party access, which is both heavily targeted by bad actors and less scrutinized than access by internal users.

## Clinical Relevance Statement

HDOs have increased risks associated with third-party access and are struggling to maintain effective cybersecurity. An online multinational survey was deployed to assess the current state of HDO third-party access cybersecurity, precautions employed, and the challenges faced. Challenges include a lack of budget and expert resources, complexity, and third-party turnover. Need exists for automated, cost-effective solutions to minimize HDO risks of third-party access and breach risk by securing remote access to privileged assets and data.

## Multiple-Choice Questions

1. Regarding the number of third parties with access to HDO networks and the annual number of cyber threats created by third-party access:
  - Both are declining.
  - Cyberthreats are increasing, but the number of third parties with network access is decreasing.
  - Both are stable with little year-to-year change.
  - Both are increasing.
  **Correct Answer** : The correct answer is option d. Both are increasing. Both the number of third parties with access to HDO networks and the number of cyber threats per year are increasing.
2. What proportion of HDOs reported having a comprehensive inventory of all third parties accessing their network?
  - 92%
  - 77%
  - 51%
  - 34%
  **Correct Answer** : The correct answer is option c. 51%. Slightly more than half of HDOs reported having a comprehensive inventory of all third parties accessing their network.

## References

[1] Riggi J Third-party cyber risk impacts the health care sector the most. Here's how to prepareAmerican Hospital Association, August 5, 2024. Third-Party Cyber Risk Impacts the Health Care Sector the Most. Here's How to Prepare. | AHA News

[2] Adams M Third-party risk management critical to protecting against cyberattackPhysicians Practice 2023;8 Mar. Gale OneFile: Health and Medicine, Accessed October 7, 2025 at:link.gale.com/apps/doc/A762612964/HRCA?u=anon∼efeb5f&sid=googleScholar&xid=b5eca4f1

[3] Sangster M The three Ps of third-party risk Cyber Security: A Peer-Reviewed Journal 2020 3 09 330 338

[4] Argaw S T Troncoso-Pastoriza J R Lacey D Cybersecurity of hospitals: discussing the challenges and working towards mitigating the risks BMC Med Inform Decis Mak 2020 20 01 146 32620167 10.1186/s12911-020-01161-7 PMC7333281

[5] Vesalainen T Most hospitals unprepared for AI-driven physical security threats, survey finds. Cybersecurity Software, June 3, 2025Most Hospitals Unprepared for AI-Driven Physical Security Threats, Survey Finds

[6] Southwick R Cybersecurity and hospitals: Big risks come from third parties. Chief Healthcare Executive, May 3, 2024Cybersecurity and hospitals: Big risks come from third parties

[7] Linden I Nearly all damaging cyber-attacks involve privileged account compromise. Cybercrime Magazine 2019Nearly All Damaging Cyber Attacks Involve Privileged Account Compromise

[8] Stone A Privileged access management is key to establishing Zero Trust. StateTech Magazine 2024Privileged Access Management: Key to Zero Trust Architecture | StateTech

[9] IBM Security Cost of a data breach report 2024. Cost of a data breach 2024 | IBM

[10] Verizon, 2023 Verizon Data Breach Investigations (DBIR). 2023 Verizon Data Breach Investigations (DBIR) Top 3 Takeaways | Proofpoint US

[11] Prevalent Mitratech The 2024 Third-Party Risk Management Study. 61% of Companies Have Been Breached by a Third Party | Prevalent

[12] Ponemon Institute The state of third-party access in cybersecurity: A Ponemon Report, 2025. The State of Cybersecurity and Third-Party Remote Access Risk

[13] Jones D Remote access tools most frequently targeted as ransomware entry points. Cybersecurity Dive, April 11, 2025Remote access tools most frequently targeted as ransomware entry points | Cybersecurity Dive

[14] Cremer F Sheehan B Fortmann M Cyber risk and cybersecurity: a systematic review of data availability Geneva Pap Risk Insur Issues Pract 2022 47 03 698 736 35194352 10.1057/s41288-022-00266-6 PMC8853293

[15] Ponemon-Sullivan, 2025. Creating a Cybersecurity Infrastructure to Reduce Third-Party and Privileged Internal Access Risks: A Global Study | Ponemon-Sullivan Privacy Report

[16] Adler S Broward Health notifies over 1.3 million individuals about October 2021 data breach. The HIPAA Journal, January 4, 2022Broward Health Notifies Over 1.3 Million Individuals About October 2021 Data Breach

[17] Steven Advanced Medical Management suffered a data breach impacted nearly 320k people. ID Strong, July 11, 2023Advanced Medical Management Got Hit by Security Breach

[18] Ewoh P Vartiainen T Vulnerability to cyberattacks and sociotechnical solutions for health care systems: Systematic review J Med Internet Res 2024 26 e46904 38820579 10.2196/46904 PMC11179043

[19] Wright J Healthcare cybersecurity and cybercrime supply chain risk management Health Econo Manag Rev 2023 4 04 17 27

[20] Rahim M J Ibn Rahim M I Afroz A Akinola O Cybersecurity threats in healthcare it: challenges, risks, and mitigation strategies J Artificial Intell General Sci 2024 6 01 438 462

[21] Alder S Study confirms increase in mortality rate and poorer patient outcomes after cyberattacks. The HIPAA Journal, September 8, 2022Study Confirms Increase in Mortality Rate and Poorer Patient Outcomes After Cyberattacks

[22] American Hospital Association Change Healthcare cyberattack underscores urgent need to strengthen cyber preparedness for individual health care organizations and as a field. January 2025Change Healthcare Cyberattack Underscores Urgent Need to Strengthen Cyber Preparedness for Individual Health Care Organizations and as a Field | AHA

[23] Alder S Ascension ransomware attack affects 5.6 million patients. The HIPAA Journal, December 20, 2024

[24] McKeon J Healthcare remains costliest industry for breaches at $7.42M. TechTarget xintelligent HealthTech SecurityJuly 30, 2025. Healthcare remains costliest industry for breaches at $7.42M | TechTarget

[25] IBM Elgin M Cost of a data breach: The healthcare industry. August 6, 2024Cost of a data breach: The healthcare industry | IBM

[26] Blanchard E E Feldman S S White M L Allen R Phillips T Brown M R Design and implementation of tabletop cybersecurity simulation for health informatics graduate students Appl Clin Inform 2024 15 05 921 927 39505007 10.1055/s-0044-1790551 PMC11540471

[27] Alhuwail D Al-Jafar E Abdulsalam Y AlDuaij S Information security awareness and behaviors of health care professionals at public health care facilities Appl Clin Inform 2021 12 04 924 932 34587638 10.1055/s-0041-1735527 PMC8481013

