# Supplementary material for: Third-Party Access Cybersecurity Threats and Precautions: A Survey of Healthcare Delivery Organizations
Source: Appl Clin Inform. 2025 Oct 30;16(5):1518–30. doi: 10.1055/a-2713-5725 (PMC12575072; doi:10.1055/a-2713-5725)
Supplement: Supplementary file 1 — Supplementary Material [file 10-1055-a-2713-5725_27356652.pdf]

Supplementary File

Cybersecurity of Third-Party Access Survey Questions

1. How familiar are you with your organization's approach to managing privileged access abuse, including processes and technologies used to secure third party, vendor and privileged end user access to your network and corporate resources? [Response options: Very Familiar; Familiar; Somewhat Familiar; No familiarity]

2. How familiar are you with your organization's approach to securing privileged access, including IT administrative access to sensitive corporate resources?

3. Please indicate your facility type [Response options: Large hospital > 500 beds; Small hospital </= 500 beds; Outpatient practice; Home health; Behavioral health; Skilled nursing facility; Other care delivery setting]

4. What is the headcount of your organization?

5. What range best describes your organization's annual IT security budget? [Response options: Less than $1 million; $1 to $10 million; $11 to $25 million; $26 to $50 million; $51 to $100 million; $101 to $250 million; $251 to $500 million; More than $500 million]

6. Does your organization have a VPAM and/or PAM solution?

7. Does your organization have a comprehensive inventory of all third parties with access to its network?

8. If no or unsure, why? Please check all that apply. [Response options: Lack of resources to track third parties; No centralized control over third-party relationships; Complexity in third-party relationships; Cannot keep track due to frequent turnover in third parties; Not a priority; Other]

9. Do you evaluate the security and privacy practices of all third parties before you engage them in a business relationship that requires providing access to sensitive or confidential information?

10. Using the following 10-point scale, please rate how effective is your organization's VPAM solution is in reducing privileged access abuse from 1 = not effective to 10 = highly effective.

11. Using the following 10-point scale, please rate how effective is your organization's PAM solution is in reducing privileged access abuse from 1 = not effective to 10 = highly effective.

12. Are third parties with access to your organization's sensitive and confidential information monitored?

13. What best describes the maturity of your organization's strategy to address privileged access risks? Please select one choice only. [Response options: Our strategy is applied consistently across the entire

organization; Our strategy is not applied consistently across the entire organization; Our strategy is ad hoc or informal; We have no formal strategy for addressing privileged access risks]

14. In the past 12 months, did your organization experience a data breach or cyberattack that involved one of your third parties/vendors accessing your organizations' network?

15. Did the data breach or cyberattack cause the misuse of the organization's sensitive or confidential information, either directly or indirectly?

16. Were these data breaches or cyberattacks the result of a third party having too much privileged access? Too much privileged access can be defined as having access to more applications and information than is needed.

17. What were the consequences of these data breaches and cyberattacks? Please select all that apply. [Response options: Severed relationships with third party/vendor; The loss or theft of sensitive and confidential information; Regulatory fines; Ransom payment; Litigation; Loss of business partners; Loss of customers; Employee turnover; Loss of revenue 22% 32% 45% 45% 36% Loss of reputation]

18. How many third parties and vendors have access to your organization's network?

19. Do you anticipate data breaches caused by third parties will increase, decrease or stay at the same level over the next 12 to 24 months?

20. Managing third party permissions and remote access to our network can be overwhelming and a drain on our internal resources. [Response options: Strongly agree; Agree; Unsure; Disagree; Strongly disagree]

21. Third parties' remote access to our network is becoming our organization's weakest attack surface. [Response options: Strongly agree; Agree; Unsure; Disagree; Strongly disagree]

22. What are the most significant barriers to reducing third-party and privileged access risks? Please select your top three choices only. [Response options: Insufficient resources or budget; Insufficient visibility of people and business processes; Insufficient assessment of risks; Difficulty in hiring and training which leads to a lack of skilled or expert personnel; Lack of leadership; Lack of oversight or governance; Complexity of compliance and regulatory requirements; Other]

23. Please check all steps taken to ensure appropriate access to high-value data assets. Please check all that apply. [Response options: Enhanced physical controls (i.e., restricted control areas); Restriction of network access; Enhanced identity and access management techniques; Ensure access entitlement is appropriate to the job function; Network segmentation/isolation; Remove access credentials when appropriate; Verification of a third party's need to have network access; Education of privileged users; Other]

24. Using the following 10-point scale, please rate how effective your organization is in mitigating remote access third-party risks. (1= not effective to 10=highly effective). [Response options: 1-2; 3-4; 5-6; 7-8; 9-10]

25. Using the following 10-point scale, please rate how effective your organization is in detecting remote access third-party risks (1=not effective to 10=highly effective). [Response options: 1-2; 3-4; 5-6; 7-8; 9-10]

26. Using the following 10-point scale, please rate your organization's effectiveness in preventing third parties from sharing credentials in the form of usernames and/or passwords (1=not effective to 10=highly effective). [Response options: 1-2; 3-4; 5-6; 7-8; 9-10]

27. Using the following 10-point scale, please rate the effectiveness of your organization in controlling third-party access to your network (1=not effective to 10=highly effective). [Response options: 1-2; 3-4; 5-6; 7-8; 9-10]

28. Using the following 10-point scale, please rate the effectiveness of your third parties in achieving compliance with security and privacy regulations that affect your organization (1=not effective to 10=highly effective). [Response options: 1-2; 3-4; 5-6; 7-8; 9-10]

29. What are the main problems your organization faces in granting and enforcing privileged user access rights? Please select your top five choices. [Response options: Takes too long to grant access to privileged users (not meeting our SLAs with the business); Too expensive to monitor and control all privileged users; Too much staff required to monitor and control all privileged users; Cannot apply access policy controls at point of change request; Granting access to privileged users is staggered (not granted at the same time); Cannot keep pace with the number of access change requests that come in on a regular basis; Lack of a consistent approval process for access and a way to handle exceptions; Difficult to audit and validate privileged user access changes; Burdensome process for business users requesting access; No common language exists for how access is requested that will work for both IT and the business; Other]

30. Do you evaluate the security and privacy practices of all third parties before you engage them in a business relationship that requires providing access to sensitive or confidential information?

31. If yes, how do you perform this evaluation? Please check all that apply. [Response options: Review written policies and procedure; Acquire signature on contracts that legally obligates the third party to adhere to security and privacy practices; Obtain indemnification from the third party in the event of a data breach; Conduct an assessment of the third party's security and privacy practices; Obtain a self-assessment conducted by the third party; Obtain references from other organizations that engage the third party; Obtain evidence of security certification such as NIST ISO 2700/27002 or SOC; Other]

32. If not, why don't you perform an evaluation? Please check all that apply. [Response options: We don't have the internal resources to check or verify; We have confidence in the third party's ability to secure information; We rely on the business reputation of the third-party; We have insurance that limits our

liability in the event of a data breach; The third party is subject to data protection regulations that are intended to protect our information; The third party is subject to contractual terms; The data shared with the third party is not considered sensitive or confidential]

33. To ensure third parties' compliance with privacy and security regulations, does your organization take any of the following steps? Please check all that apply. [Response options: Identify and categorize third-party vendor and partner access needs; Perform access assessments for each vendor and partner; No vendor-supplied security parameters or default passwords; Implement least privileged access; Insist on unique user access credentials; Encrypt transmissions for all open or public networks; Track and monitor all access to network resources and critical data; Capture detailed audit logs of each support session; Install and maintain a firewall configuration to protect data; Develop secure application and system implementation; Protect all systems against malware and regularly monitor anti-virus protections; Restrict physical access; Other]

34. Approximately, how many hours each week are spent analyzing and investigating the security of third party and privileged access? Please estimate the aggregate hours of the IT security team? [Response options: Less than 5 hours; 5 to 10; 11 to 25; 26 to 50; 51 to 100; 101 to 250; 251 to 500; More than 500]
